# Efficacy of Bracing on Thoracic Kyphotic Angle and Functionality in Women with Osteoporosis: A Systematic Review

**DOI:** 10.3390/medicina58060693

**Published:** 2022-05-24

**Authors:** Beatriz Sánchez-Pinto-Pinto, Carlos Romero-Morales, Daniel López-López, Carmen de-Labra, Guillermo García-Pérez-de-Sevilla

**Affiliations:** 1 Hospital de Emergencias Enfermera Isabel Zendal, Physical Medicine and Rehabilitation Unit, 28055 Madrid, Spain; bsanchezpinto@gmail.com; 2 Faculty of Sports Sciences, Department of Physiotherapy, Universidad Europea de Madrid, 28670 Madrid, Spain; carlos.romero@universidadeuropea.es; 3 Research, Health and Podiatry Group, Department of Health Sciences, Faculty of Nursing and Podiatry, Universidade da Coruña, Industrial Campus of Ferrol, 15403 Ferrol, Spain; daniel.lopez.lopez@udc.es; 4 NEUROcom, Centro de Investigaciones Científicas Avanzadas (CICA), Instituto de Investigación Biomédica de A Coruña (INIBIC), Faculty of Nursing and Podiatry, University of A Coruna, 15001 La Coruña, Spain; c.labra@udc.es

**Keywords:** osteoporosis, bracing, spinal orthosis, kyphosis

## Abstract

*Background and purpose:* Osteoporotic hyperkyphosis is associated with adverse outcomes, such as fatigue, back pain, or reduced back extensor strength, with a negative impact on functionality and quality of life. The purpose of this review is to assess the effectiveness of spinal orthosis on these adverse effects. *Methods:* A systematic review following the PRISMA guidelines was performed. Inclusion criteria were (1) women with osteoporosis; (2) randomized controlled trials only; and (3) type of intervention: spinal bracing. Exclusion criteria were (1) article not written in English; (2) full-text not available; and (3) no kyphosis assessment. Quality-of-life variables such as back pain, functional variables such as back extensor strength, and osteoporotic-related variables such as lumbar spine bone mineral density were extracted and recorded before and after the intervention. The characteristics of the intervention programs were also extracted and recorded. The characteristics of studies, interventions, and participants are summarized in a table. Then, the revised Cochrane risk-of-bias tool for randomized trials (RoB 2) was used to assess the quality of the studies. *Results and Discussion:* Four randomized controlled trials with a low risk of bias were included (*n* = 326 women with osteoporosis, aged 51–93 years). Interventions consisting of wearing a dynamic hyperextension orthosis for at least two hours per day for six months improved functionality, mobility, back extensor strength, respiratory function, and reduced the thoracic kyphosis angle. *Conclusions:* Spinal orthosis, especially dynamic hyperextension braces, seems effective in improving the adverse outcomes of osteoporotic hyperkyphosis. It does not seem necessary to wear the orthosis during all daily activities.

## 1. Clinical Implications

Spinal orthosis seems to be effective in reducing the thoracic kyphosis angle, but there is a lack of evidence about this topic.

Wearing a dynamic hyperextension brace for two hours per day for at least six months may improve functionality and reduce the thoracic kyphosis angle in older women with hyperkyphotic osteoporosis.

The clinical relevance of this study is that it does not seem necessary to wear the orthosis during all daily activities.

## 2. Introduction

Osteoporosis is the most common chronic metabolic bone disease, characterized by low mineral bone mass, structural deterioration of the bone tissue, decreased cortical thickness, increased porosity, and altered bone microvasculature. These characteristics lead to a lower bone quality and, therefore, higher bone fragility and susceptibility to fractures [1,2,3].

Osteoporosis is more prevalent in Caucasians, older people, and women [4]. With an aging population and longer life span, osteoporosis is becoming an epidemic as more than 200 million people worldwide are suffering from the disease, which affects one in three women and one in five men over the age of 50 years [4]. In Europe and the United States, 30% of women have osteoporosis and about 40% of post-menopausal women and 30% of men will experience an osteoporotic fracture in their lifetime [5].

In fact, osteoporotic fractures are a major contributor to medical care costs and a main cause of disability. The social burden of fractures will increase throughout the world as the population ages [6]. The most prevalent osteoporotic fractures are in the wrist, hip, proximal humerus, and spine. It is a silent disease until fractures occur, which causes important secondary long-term pain and, in some cases, death [7].

Although a fracture is commonly the first sign of osteoporosis, some older people develop the characteristic of a stooped posture, which is caused by vertebral micro-fractures that could result in anterior height reduction in vertebral bodies, and thus thoracic hyperkyphosis [8].

Thoracic hyperkyphosis is associated with adverse outcomes, such as persistent fatigue and back pain, reduced back extensor strength, loss of height, deformity, immobility, depression, and even reduced pulmonary function, with a negative impact on functionality and quality of life [9].

Spinal orthosis seems to help in reducing excess flexion and correcting posture by promoting a neutral thoracic and lumbar alignment [10,11]. Reducing thoracic hyperkyphosis leads to an improved balance, trunk muscles activation, and functionality, therefore reducing the risk of falls and further fractures [12]. Additionally, spinal orthosis increases sensory feedback and postural control [13].

However, few randomized controlled trials have analyzed the effects of wearing a spinal orthosis for thoracic hyperkyphosis, despite the fact that is a cost-effective treatment. The aim of this review is to assess the effectiveness of wearing a spinal orthosis on the thoracic kyphosis angle, quality of life, and functionality in older women with osteoporotic hyperkyphosis.

## 3. Methods

This systematic review was performed according to the Preferred Reporting Items for Systematic Reviews and Meta-analyses (PRISMA) statement [14]. This systematic review was registered in PROSPERO (International prospective register of systematic reviews) on 4 November 2021, with the registration number CRD42021283480.

### 3.1. Data Source

Studies were identified in PubMed, Rehabilitation and Sports, Scopus, Web of Science, and CINAHL and were published from the earliest time point until 1 May 2022. The search terms were: “Osteoporosis AND (bracing OR back orthosis OR spinal orthosis) AND kyphosis”. Grey literature (e.g., abstracts, conference proceedings, and editorials) and reviews were excluded. After the duplicates were removed, two authors (BSPP and GGPS) independently screened the titles and abstracts and then evaluated the full texts of potentially relevant studies. Disagreements were resolved through consultation with a third review author (CRM). Studies were eligible for inclusion if they met the eligibility criteria. All excluded studies were recorded, and the reasons for exclusion were provided.

### 3.2. Inclusion and Exclusion Criteria

The criteria for inclusion were (1) population: women with osteoporosis; (2) type of study: randomized controlled trials only; and (3) type of intervention: wearing a spinal orthosis. The exclusion criteria were (1) article not written in English; (2) full text not available; and (3) no kyphosis assessment.

### 3.3. Data Extraction

Full-text articles matching the inclusion criteria were retrieved for all studies. They were electronically stored and systematically reviewed. Descriptive outcomes and results from the intervention were extracted and recorded using a spreadsheet by two independent investigators (BSPP and GGPS). In case of disagreement, a third investigator (CRM) assessed the study, and the disagreement was resolved by consensus. The characteristics of the intervention programs were also extracted and recorded.

### 3.4. Data Synthesis

Characteristics of studies, interventions, and participants are summarized in Table 1. Revised Cochrane risk-of-bias tool for randomized trials (RoB 2) [15] was used to assess the following characteristics: (1) bias arising from the randomization process; (2) bias due to deviations from intended interventions; (3) bias due to missing outcome data; (4) bias in the measurement of the outcome; and (5) bias in the selection of the reported result. Two investigators (BSPP and GGPS) performed separate assessments of risk of bias. In case of disagreement, a third investigator (DLL) assessed the study, and the disagreement was resolved by consensus.

## 4. Results

### 4.1. Search Outcome

**Figure 1:**
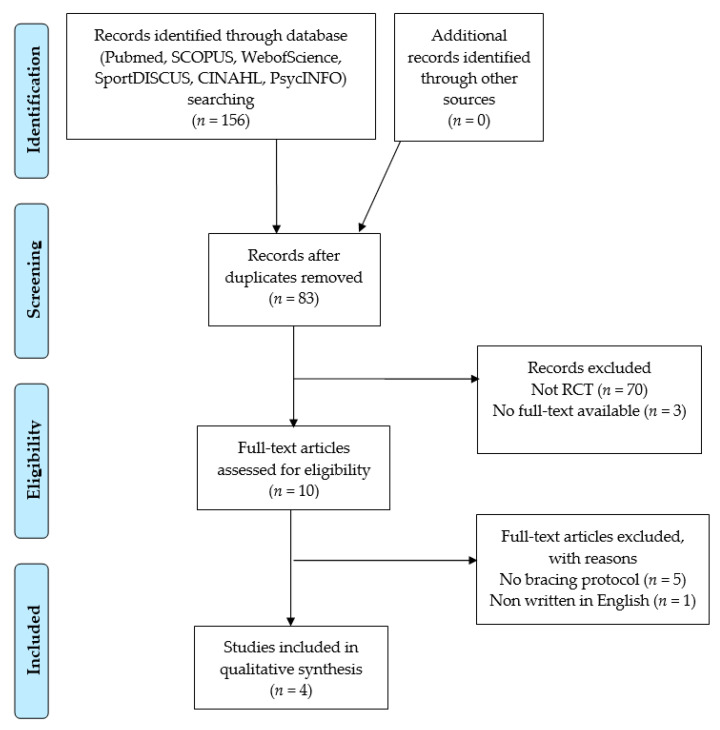
Preferred reporting items for systematic reviews and meta-analyses flow diagram for study selection.

The search strategy identified 156 articles from electronic databases. Following removal of duplicates, 84 articles were initially screened via their titles and abstracts, and 11 were identified as potentially relevant. The full-text examination further excluded seven studies, leaving four studies for inclusion in this analysis, all of which were randomized controlled trials (Figure 1).

### 4.2. Characteristics of the Included Studies

*Participants:* In the four studies analyzed, a total of 326 older women with osteoporosis aged 51–93 years were analyzed. Participant demographics, intervention characteristics, and outcomes are summarized in Table 1.

*Interventions*: Bracing interventions were conducted for 6 to 12 months, using a Spinomed orthosis (two studies), Spinomed active orthosis (one study), an activating spinal orthosis (one study), and a dynamic hyperextension brace (one study). The participants wore the orthosis for 2 to 12 h per day.

*Results:* Variables assessed were categorized into three groups: (i) Quality-of-life variables—limitations of daily living, back pain; (ii) functional variables—back extensor strength, abdominal flexor strength, forced expiratory volume in 1 sec, and vital capacity; and (iii) osteoporotic-related variables—thoracic kyphosis angle, body height, lumbar spine bone mineral density, and T-score.

### 4.3. Quality Assessment of Study Methodology

**Figure 2:**
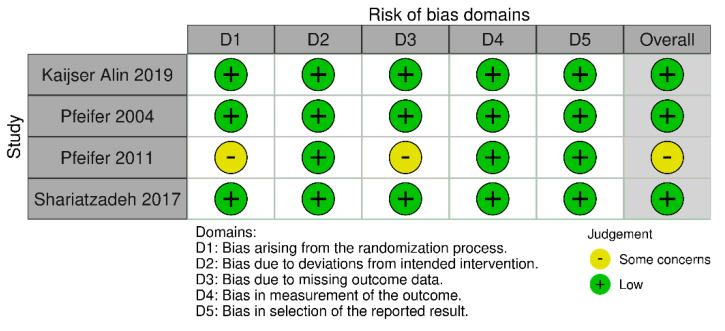
Risk of bias summary of the studies included.

The risk-of-bias analysis revealed that one (25%) study presented some concerns about the randomization process and m of the missing outcome data. Overall, 75% of the included studies presented a low risk of bias (Figure 2).

### 4.4. Effect of the Intervention

*Quality of life variables:* In the study by Pfeifer et al., 2004 and 2011 [12,16] showed improved variables for the limitations of daily living and reduced back pain, while the study by Kaijser Alin et al. showed no changes [17] (Table 1).

*Functional variables*: Pfeifer et al., 2004 and 2011 [12,16] showed significant improvements in back extensor strength, abdominal flexor strength, forced expiratory volume in 1 s, and vital capacity (Table 1).

*Osteoporotic-related variables:* The four studies included in this review analyzed the thoracic kyphosis angle, three of which found a significant decrease [12,16,18], while Kaijser Alin et al. [17] observed no significant changes. Pfeifer et al., 2004 and 2011 [12,16] obtained a significant increase in body height and Shariatzadeh et al. [18] observed a significant increase in the lumbar spine bone mineral density but not in the lumbar spine T-score (Table 1).

**Table 1:** Participants demographics, outcomes assessed, intervention characteristics, and results of included studies.

Authors, Year	Participants	Intervention	Results
Kaijser Alin et al., 2019 [17]	Women with osteoporosis aged 76 years *n* = 96 IG1: *n* = 31; IG2: *n* = 31; CG: *n* = 34	IG1: Activating spinal orthosis two hours per day IG2: Physical exercise Duration: Six months	IG1: ↔Back pain; ↔Back extensor strength; ↔Thoracic kyphosis angle IG2: ↔Back pain; ↔Back extensor strength; ↔Thoracic kyphosis angle
Kaijser Alin et al., 2021 [19]	Women with osteoporosis aged 76 years *n* = 31	A six-month post-intervention follow-up of women who voluntarily continued to wear the spinal orthosis	↔Back extensor strength
Shariatzadeh et al., 2017 [18]	Women with hyperkyphotic osteoporosis (Cobb angle 50°–65°) aged 63.3 ± 10.8 years *n* = 60; IG: *n* = 30; CG: *n* = 30	IG: Dynamic hyperextension brace 12 h per day + spinal hyperextension exercises + vitamin D + calcium + alendronate Duration: 12 months	IG: ↓Thoracic kyphosis angle (−7.5°); ↑Lumbar spine bone mineral density; ↔T-score
Pfeifer et al., 2011 [16]	Women with hyperkyphosis (Cobb angle ≥ 60°) and ≥1 vertebral fracture, aged 72 years *n* = 108 IG1: *n* = 36; IG2: *n* = 36; CG: *n* = 36	IG1: Spinomed orthosis two hours per day IG2: Spinomed active orthosis two hours per day Duration: Six months	IG1: ↓Thoracic kyphosis angle (−7.9°); ↑Back extensor strength; ↑Abdominal flexor strength; ↑ Forced expiratory volume in 1 s; ↑Vital capacity; ↓Back pain; ↓ Limitations of daily living; ↑Body height (+5.3 cm) IG2: ↓Thoracic kyphosis angle (−8.1°); ↑Back extensor strength; ↑Abdominal flexor strength; ↑ Forced expiratory volume in 1 s; ↑Vital Capacity; ↓Back pain; ↓Limitations of daily living; ↑Body height (+6.1 cm)
Pfeifer et al., 2004 [12]	Women with hyperkyphosis (Cobb angle ≥60°) and ≥1 vertebral fracture, aged 72 years *n*= 62 IG: *n* = 31; CG: *n* = 31	IG: Thoracolumbar orthosis Spinomed (Medi-Bayreuth, Bayreuth, Germany) two hours per day + vitamin D + calcium + biphosphonate. Duration: Six months	IG: ↓Thoracic kyphosis angle (−4.2°); ↑Back extensor strength; ↑Abdominal flexor strength; ↑Forced expiratory volume in 1 s; ↑Vital capacity; ↓Back pain; ↓Limitations of daily living; ↑Body height (+5.8 cm)

Abbreviations: IG, intervention group; CG, control group.

## 5. Discussion

The main findings from this review are that older women with osteoporosis who used a spinal orthosis for at least two hours a day for six months achieved a significant reduction in thoracic kyphosis angle. They also achieved significant improvements in terms of back pain, back extensor strength, pulmonary function, and quality of life. However, few studies were included in this review, so these results should be interpreted with caution.

In the study conducted by Kaijser Alin et al., wearing and activating spinal orthosis for two hours a day for six months were not effective in reducing the thoracic kyphosis angle, compared to the group control. However, in a six-month post-intervention follow-up, the participants who voluntarily continued to use the spinal orthosis maintained their increase in back extensor muscle strength [19]. The three other studies achieved a significant reduction in the thoracic kyphosis angle by wearing a thoracolumbar orthosis Spinomed two hours a day for six months (Pfeifer et al., 2004, Pfeifer et al., 2011), a thoracolumbar active orthosis Spinomed two hours a day for six months (Pfeifer et al., 2011), and a dynamic hyperextension brace twelve hours a day for 12 months (Shariatzadeh et al., 2017). Therefore, wearing a spinal orthosis for two hours a day for six months seems effective for reducing the thoracic kyphosis angle (mean reductions between −7.5° and −8.1°) and promoting a neutral thoracic and lumbar alignment.

A significant reduction in the thoracic kyphosis angle was associated with an improvement in back extensor strength, abdominal flexor strength, back pain, limitations of daily living forced expiratory volume in 1 sec, and vital capacity as previously described by other authors [9]. In the studies conducted by Pfeifer et al. in 2004 and 2011, the intervention group also increased their body height > 5 cm. Therefore, reducing the thoracic kyphosis angle may lead to an increase in functionality, respiratory function, and quality of life. In contrast, Shariatzadeh et al. did not show any changes in back pain, back extensor strength, nor the thoracic kyphosis angle.

Only Shariatzadeh et al. analyzed the lumbar spine bone mineral density and the T-score, obtaining significant improvements in lumbar spine bone mineral density but not in T-score, so these improvements do not seem clinically significant. More studies are needed to analyze these relevant variables, as the start of the pharmacological treatment usually depends on the T-score (or the Z-score for younger patients) [20].

Kaijser Alin et al. compared wearing a spinal orthosis with back strength training, finding no differences between both interventions in back pain, thoracic kyphosis angle, and back extensor strength. This study detailed the entire exercise protocol, which could be very useful in future clinical trials. In the study carried out by Shariatzadeh et al., the combination of bracing and exercise was more effective than exercise alone; although, in this case, the exercise protocol was not sufficiently detailed. However, because Shariatzadeh et al. combined exercise with bracing, it is not known if bracing alone could have achieved the same results.

The main limitation of this review is the lack of high-quality studies analyzing the effects of wearing a spinal orthosis in people with osteoporosis, so the quality of the evidence is moderate, and more randomized controlled trials with a low risk of bias are necessary. The strength of this study is that two hours a day during six months of orthotic treatment seems to be sufficient for obtaining clinically significant results on the adverse effects of osteoporosis, so it does not seem necessary to wear the orthosis during all daily activities, as is commonly prescribed. Furthermore, reducing the thoracic kyphosis angle was associated with improved trunk muscles activation and functionality, which could reduce the risk of falls and further fractures [21].

## 6. Conclusions

This review summarizes the current knowledge on the effectiveness of wearing a spinal orthosis on the thoracic kyphosis angle, quality of life, and functionality in older women with osteoporotic hyperkyphosis. Older women with osteoporosis wearing a spinal orthosis two hours a day for six months may achieve significant reductions in thoracic kyphosis angles, which is associated with improved functionality, quality of life, and respiratory function. Overall, this could reduce the risk of falls and fractures by wearing the orthosis two hours a day.

## Data Availability

The datasets used and/or analyzed during the current study are available from the corresponding author on reasonable request.
